# Remedies and Their Uses

**Published:** 1907-01-05

**Authors:** 


### Remedies and their Uses.

**Cardiac Tonics.**

Regarded from one point of view no class of drugs are more certain in their beneficent effect than those employed to strengthen and slow the beat of the heart, but from another point of view no class is less certain in its action or more elusive chemically. The apparent paradox is explained by the fact that whereas the active principles of digitalis and its allies are most potent and certain in their action, the pharmacopœial preparations are liable to vary considerably in strength and composition, and pharmacologists are not yet absolutely decided as to the relative values of their various constituents. According to some recent experiments by Kakowski [1] the most typical results on the excised mammalian heart are obtained when fresh preparations of the digitalis leaves are used, the various glucosides (digitoxin, digitalin, and digitalein) giving a similar but not such a complete result. It is now generally held that digitoxin is the most important and active glucoside, and, considering the great variability of the infusion, the use of a pure drug, especially if it can be kept conveniently without decomposition, presents enormous advantages from the clinical point of view. Merck has succeeded in preparing a pure crystalline preparation of digitoxin, which has all the therapeutic properties of digitalis leaves, but it is very insoluble in water. The aqueous extract of the leaves, however, appears to contain digitoxin in some soluble form, and Cloetta has recently claimed by a somewhat elaborate process to have succeeded in preparing this soluble digitoxin in a pure form. The preparation, which is known as

**Digalen,**

is an amorphous white powder, supplied in aqueous solution; 25 per cent. glycerine is added to the solution, which is sent out in small bottles. Each cubic centimetre contains .3 milligramme of digitalin, and a graduated pipette is supplied with each bottle to facilitate dosage. The maximum dose is said to be 4 c.c., and the ordinary adult dose 1 c.c. of the solution (17 minims). It is manufactured by Hoffmann, La Roche and Company, of Basel. Freund considers this the best digitalis preparation on the market. It can owing to its solubility be given intravenously—an operation which is generally not difficult after a little practice. He has not observed the unpleasant effects (nausea, vomiting, diarrhœa) sometimes occurring after digitalis is given by the mouth, and thinks that the more serious symptoms of cardiac arrest—dyspnœa and convulsions—are not to be feared owing to the non-cumulative action of digalen. He has found it particularly valuable in asthma, its action being very prompt. Vlach [3] considers it a good preparation, and finds that it only fails in a few cases, where digitalis in any form is useless. The maximum dose he puts at 1 c.c. daily. He thinks that administration by the mouth is the best method, unless the drug is badly borne by the stomach. Vlach believes that a cumulative action may occur. Weinberger [4] employs digalen in 1 c.c. doses two or three times daily. Intravenously he gives 2-5 c.c. He points out the value of the drug when an immediate and powerful effect is desired. Hochheimer [5] calls attention to the fact, noted also by others, that digalen if used subcutaneously causes considerable pain and infiltration, in this was resembling crystalline digitoxin. It may, however, be necessary to use this method if the drug is not tolerated by the stomach. By the mouth he gives it mixed with peppermint water, while Grassmann advises a little syrup or sweet wine as a vehicle. The latter author thinks digalen well tolerated as a rule even by those whose stomachs are easily upset. All observers agree that digalen is a powerful diuretic, but if it is desired to increase this effect it may be combined with 1 gramme (15 grains) of diuretin. Hersig [6] has compared the leucocytosis produced by the various digitalis compounds, and finds digalen and amorphous digitonin give more or less marked leucocytosis during twenty-four hours, but that with digitalin this reaction was very slight, and with crystalline digitonin absent.

[1] Archiv. Intern. de Pharm. et de Thérap., xv., 1905. [2] Thérap. Monatsh, xix. 12, 1905. [3] Prague M. W. xxxi. 4, 1906. [4] Centralbl. f. Innere. Med. xxvi. 27. 905. [5] Ibid., 22. 1905. [6] Arch. f. Exper. Path. a. Pharm., lii., 2, p. 177. 1905.

